# Effects of Bulk LPSO Phases on Mechanical Properties and Fracture Behavior of As-Extruded Mg-Gd-Y-Zn-Zr Alloys

**DOI:** 10.3390/ma16237258

**Published:** 2023-11-21

**Authors:** Dongjie Chen, Ting Li, Zhaoqian Sun, Qi Wang, Jiawei Yuan, Minglong Ma, Yonggang Peng, Kui Zhang, Yongjun Li

**Affiliations:** 1 School of Architecture and Civil Engineering, Huanghuai University, Zhumadian 463000, China; chendjie@126.com (D.C.);; 2 Guobiao (Beijing) Testing & Certification Co., Ltd., Beijing 100088, China; 3 China United Test & Certification Co., Ltd., Beijing 100088, China; 4 State Key Laboratory of Nonferrous Metals and Process, GRINM Group Co., Ltd., Beijing 100088, Chinazhkui@grinm.com (K.Z.); lyj@grinm.com (Y.L.); 5 GRIMAT Engineering Institute Co., Ltd., Beijing 101407, China

**Keywords:** Mg-Gd-Y-Zn-Zr alloy, long-period stacking-ordered (LPSO) phases, fracture behavior, mechanical properties

## Abstract

Despite the consensus on the constructive effect of LPSO (long-period stacking-ordered) phases, the true effect of bulk LPSO phases on strengthening and toughening in deformed magnesium alloys is still controversial. This article, which introduces the alloys Mg-8Gd-4Y-0.6Zn-0.5Zr, without bulk LPSO phases, and Mg-8Gd-4Y-1.6Zn-0.5Zr, containing bulk LPSO phases, details a systematically comparative analysis conducted to clarify the true contribution of bulk LPSO phases to the properties of as-extruded alloys. The results indicate that bulk LPSO phases significantly improve strength by refining grain sizes remarkably. But contrary to expectations, bulk LPSO phases themselves only provide a small strengthening effect and deteriorate plasticity, ascribed to the poor compatible plastic deformation of bulk LPSO phases. More importantly, this work may offer new insights into the strengthening and toughening of LPSO phases for further research and engineering applications of this series of alloys. Additionally, an example of a design strategy for Mg-Gd-Y-Zn alloys with superior strength and excellent plasticity is proposed at the end of this article.

## 1. Introduction

The demands for lower energy consumption and higher flexibility in the automobile and aerospace industries have triggered a strong interest in using magnesium alloys, especially high-performance magnesium alloys, due to their advantages in terms of weight reduction and potential as load-bearing structure materials [1,2,3]. However, on account of the characteristics of hexagonal close-packed structures, basal slip systems are predominantly activated at room temperature, which means these structures cannot reach the requirements of the Mises plastic deformation criterion and lead to poor ductility [4,5]. In addition, the precipitated phases, such as β′ and β_1_ in high-strength magnesium rare-earth (RE) alloys [6,7,8], will further deteriorate the plasticity significantly, despite effectively improving the strength of the alloys.

A recent strategy for improving mechanical properties significantly is to introduce LPSO phases in magnesium alloys by adding RE elements and transition metal (TM) elements [9]. The typical crystal structures of LPSO phases are 18R and14H structures, in which the RE/TM atoms are periodically segregated into four specific layers of close-packed planes with face-centered-cubic (FCC)-like stacking [10,11]. Kawamura et al. [12] first reported a rapidly solidified Mg97Zn1Y2 alloy containing ~24 vol% of an LPSO phase, exhibiting an extremely high yield strength of over 600 MPa and a retaining elongation of 5%. Thereafter, several Mg-RE-Zn alloys with superior tensile strength, of over 500 MPa, and outstanding elongation properties, near 10%, were obtained via hot extrusion followed by an aging treatment [13,14]. To elucidate the physical origin underlying these excellent mechanical properties, the plastic deformation and mechanical properties of LPSO phases have been extensively studied. It was found that the deformation mode of an LPSO phase strongly depends on the loading direction. Basal slip is the most commonly noted in LPSO phases due to having the lowest critical shear stress (CRSS) [15,16]. Additionally, in the case where pressure stress is applied parallel to the basal plane, unique kink bands may be formed [17], and non-basal slip can be operative in the LPSO phase when stretched parallel to the basal plane [18]. These deformation modes of LPSO phases present a potential to improve plasticity. As for strength, Young’s modulus and the shear modulus of a single-phase LPSO crystal were detected in much higher states than those of pure Mg [19,20], allowing LPSO phases to act as hard phases [19] and hindering dislocation slip. In addition, the formed kink bands were found to act as effective obstacles to the motion of dislocations, contributing to the strengthening of the LPSO phases [21]. As a result, LPSO phases are believed to contribute to the ductility and strength of Mg-RE-Zn alloys simultaneously.

However, in fact, there is still controversy over the true contribution of LPSO phases when it comes to the mechanical properties of experimental alloys. Although some studies [22,23,24,25] have attributed their high plasticity to a combination effect such as that of fine grains and LPSO phase deformation, there are still studies [20,26] proposing that LPSO phases destroy the plasticity of alloys while strengthening them with fiber reinforcement. The main reason for this controversy is that the excellent mechanical properties of the alloys are usually the result of a combination of factors such as grain size, texture, LPSO phases, and other precipitations. To make matters more complicated, LPSO phases typically come in two forms: an irregular bulk form at grain boundaries and an intragranular lamellar form [27,28]. Thus, it is difficult to separate the true contribution of LPSO phases to properties from all the strengthening effects. In order to elucidate the true contribution of LPSO phases to mechanical properties, it was important to carry out a comparable study of the contributions of different factors to the strength and plasticity in alloys with and without LPSO phases. In our previous research [29], we discovered that lamellar LPSO phases exhibit a weak strengthening effect when basal dislocation slip dominates and cracks extend along the basal plane of the Mg matrix. A similar phenomenon was found in a study by Zheng et al. [30]. On this basis, further comparable studies are needed to determine the true contribution of bulk LPSO phases.

Therefore, for this paper, a Mg-8Gd-4Y-0.6Zn-0.5Zr alloy free of bulk LPSO phases and a Mg-8Gd-4Y-1.6Zn-0.5Zr alloy containing bulk LPSO phases were prepared, and a comparative analysis was conducted to discuss and explain the role of bulk LPSO phases in the mechanical properties and fracture behavior of the alloys, potentially providing essential guidance for high-performance magnesium alloy design.

## 2. Materials and Methods

Direct chill casting was used to create ingots of Mg-Gd-Y-Zn-Zr alloys that were 400 mm in diameter and 800 mm in length. The actual chemical compositions of the alloys were analyzed using inductively coupled plasma atomic emission spectroscopy (ICP-AES; Agilent-5110, Agilent, Santa Clara, CA, USA) and are listed in Table 1. According to our previous research [31], cylindrical samples, made from ingots with diameters and heights of 115 mm and 260 mm, respectively, were homogenized at 500 °C for 48 hours in a muffle furnace to eliminate nonequilibrium second phases and obtain two alloys, Mg-8Gd-4Y-0.6Zn-0.5Zr without bulk LPSO phases (referred to as 0.6Zn alloy) and Mg-8Gd-4Y-1.6Zn-0.5Zr containing bulk LPSO phases. The alloys were then cooled down to 450 °C at a rate of 0.7 °C/min and kept at that temperature for two hours. And extrusion at 450 °C, with an extrusion ratio of 23:1 and a ram speed of 0.4 mm/s, followed by water quenching, was used to create extrusion rods with a diameter of 25 mm.

The microstructures of the alloys were observed using an optical microscope (OM, Axiovert-200MAT, Zeiss, Jena, Germany) and a scanning electron microscope (SEM, JSM-7610F, Jeol, Tokyo, Japan), equipped with an X-Max 80 energy dispersive spectrometer (EDS) and Symmetry electron backscatter diffractometer, and a transmission electron microscope (TEM, Talos-F200X, ThermoFisher, Waltham, MA, USA). The texture was analyzed using an EBSD system operating at 25 kV, and the data were analyzed using OIM Analysis (TSL OIM 8.0) software. The tensile specimens, which had a gauge length of 25 mm and a diameter of 5 mm, were machined from the as-extruded rods parallel to the direction of extrusion. These specimens were then tested using a universal material testing machine (SANS, CMT5504, Rockville, MD, USA) with a crosshead speed of 2 mm/min at ambient temperature. For each alloy, at least three specimens were used to obtain a consistent stress–strain curve.

## 3. Results and Analysis

### 3.1. Microstructure of the As-Extruded Alloys

**Figure 1:**
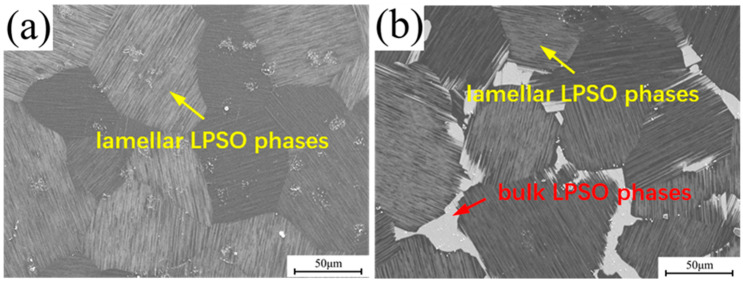
SEM images of the (**a**) 0.6Zn alloy and (**b**) 1.6Zn alloy before extrusion, which were homogenized at 500 °C for 48 h, followed by being cooled in a furnace to 450 °C and held at this temperature for 2 h.

Figure 1 depicts SEM images of the representative microstructures of the two alloys after the process of homogenization and subsequent furnace cooling. In the 0.6Zn alloy, nearly all eutectic phases have dissolved into the α-Mg matrix. However, in the 1.6Zn alloy, there are significant amounts of bulk LPSO phases present at grain boundaries, especially at the triple junctions. And dense lamellar LPSO phases or stacking faults (SFs), which precipitate from the α-Mg phases during furnace cooling, have been observed appearing parallel to each other within a grain. Furthermore, the average grain sizes of the 0.6Zn and 1.6Zn alloys were measured to be approximately 88 μm and 92 μm, respectively.

**Figure 2:**
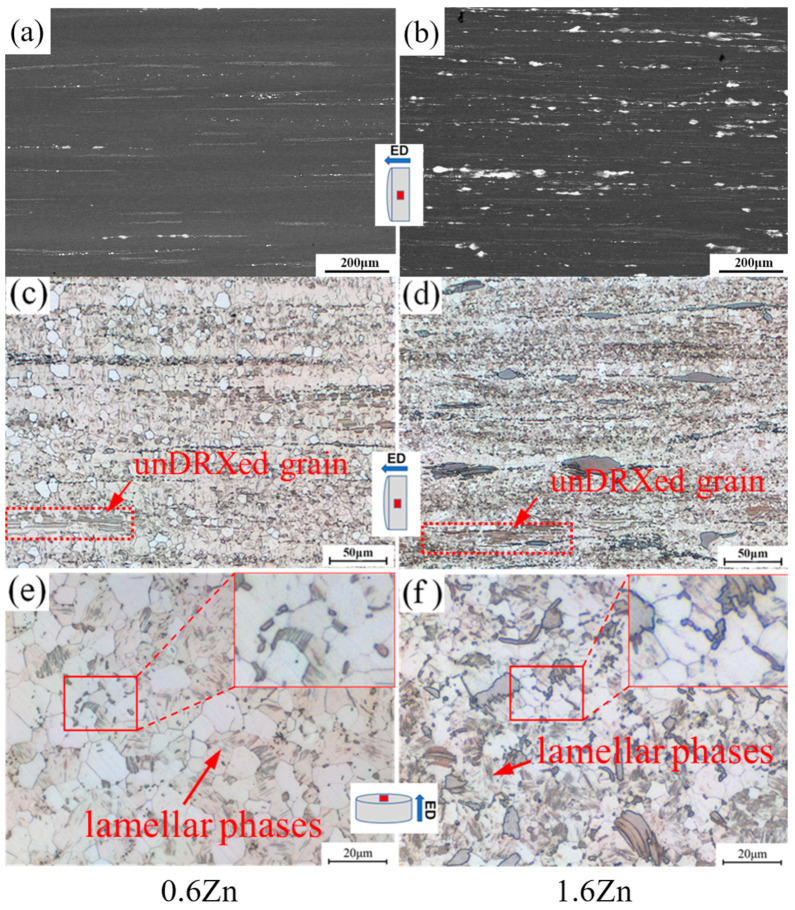
Micrographs of the as-extruded alloys: (**a**,**b**) SEM micrographs and (**c**–**f**) optical micrographs (**a**–**d**) along extrusion direction and (**e**,**f**) perpendicular to the extrusion direction.

Figure 2a,b show SEM micrographs taken along the extrusion direction (ED). The contrast varies depending on the chemical composition, with the α-Mg phases appearing black and the other phases (mostly the LPSO phase) appearing white. The bulk LPSO phases in the 1.6Zn alloy are noticeably distorted after extrusion, with the majority being elongated along the ED and some even being shattered. The optical micrographs in Figure 2c,d reveal that fine dynamically recrystallized (DRXed) grains were obtained in both of the extruded alloys, while the remaining few grains that did no undergo dynamic recrystallization are also significantly refined, with lamellar phases parallel to the ED. As shown in Figure 2e,f, the lamellar phases generated during the furnace-cooled process disappeared in the DRXed grains, whereas some new finer lamellar phases were precipitated in some DRXed grains. Furthermore, fine particles are also present in both extruded alloys, with the majority of them being located near grain boundaries, as shown in the red boxes.

**Figure 3:**
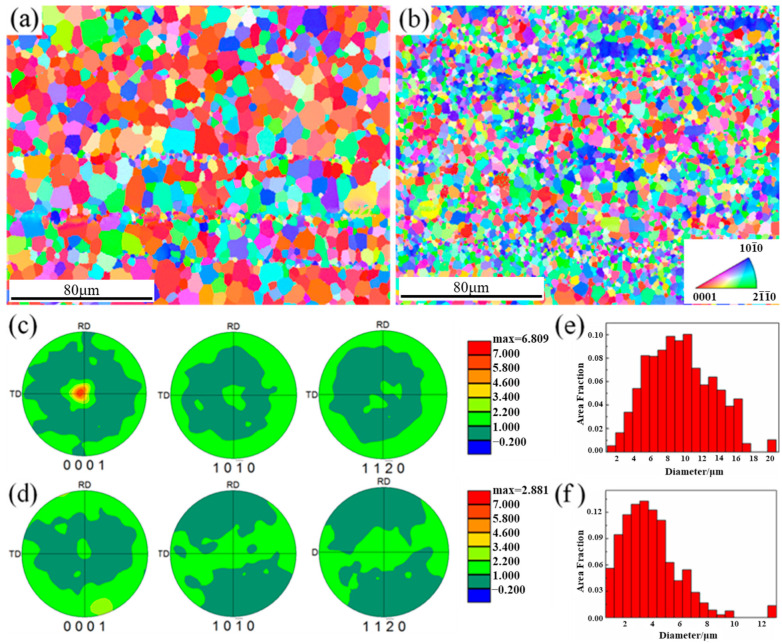
The EBSD analysis results taken from the as-extruded (**a**,**c**,**e**) 0.6Zn alloy and (**b**,**d**,**f**) 1.6Zn alloy. (**a**,**b**) IPF maps taken perpendicular to the extrusion direction, (**c**,**d**) pole figures, and (**e**,**f**) grain size distribution.

Figure 3 shows the results of EBSD analysis for extruded alloys. Similarly, almost complete dynamic recrystallization occurred in both alloys. The DRXed grains in the 0.6Zn alloy are noticeably larger than those in the 1.6Zn alloy, as shown in Figure 3a and b, respectively. The grain size distribution images reveal that the grain sizes in the 0.6Zn alloy are mostly distributed within 20 μm, with an average size of 8.9 μm. In contrast, the grain sizes in the 1.6Zn alloy are mainly distributed within 10 μm, with an average size of 4.0 μm (Figure 3e,f). According to the pole figure in Figure 3c,d, the 0.6Zn alloy exhibits a typical basal texture with {0001} basal planes parallel to the ED, making it much stronger than the 1.6Zn alloy. This difference in intensity may be attributed to the dynamic recrystallization promoted by LPSO phases during the deformation process. In fact, it has been proven that the ability of the LPSO phases to promote dynamic recrystallization depends on the deformation conditions, which impact the coordinated deformation capability of the LPSO phases. Although some studies have suggested that dynamic recrystallization is inhibited by the coordinated deformation of LPSO phases, such as kinking or dislocation gliding [32], it was difficult for LPSO phases to continuously coordinate plasticity and release dislocations in this experiment, as the extrusion ratio was much higher than that in most experiments. Therefore, the dislocations piled up at the interfaces of LPSO phases, which can lead to a significant promotion of dynamic recrystallization. As a result, the grain sizes were finer, and a more random texture was obtained in the 1.6Zn alloy [14].

**Figure 4:**
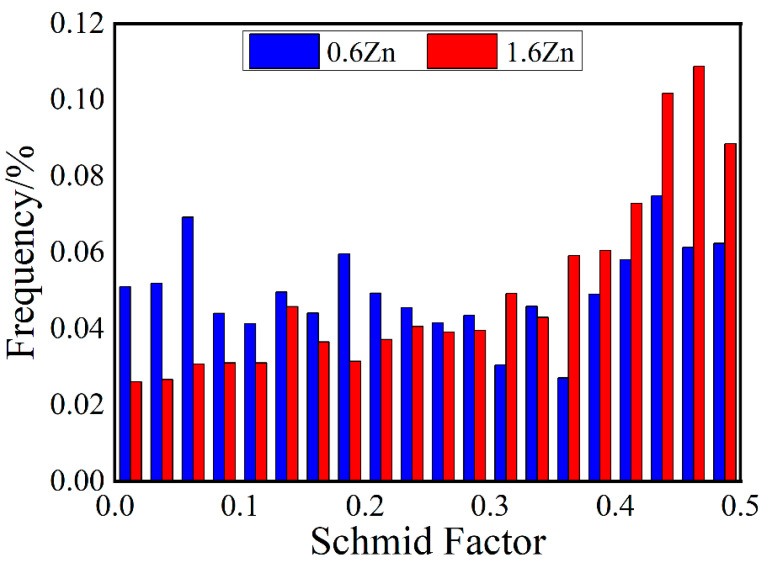
Statistics of Schmid factors of basal (0001) <11$\bar{2}$0> slips for the as-extruded 0.6Zn and 1.6Zn samples when loaded in extrusion direction.

Figure 4 shows the (0001) <11$\bar{2}$0> Schmid factor distributions for the as-extruded alloys when loaded in the extrusion direction. The 1.6Zn alloy exhibits a tendency to distribute towards higher values compared to the 0.6Zn alloy. This indicates that basal slip is more likely to be activated during a tensile test in the 1.6Zn alloy, which benefits plastic deformation when loaded along the extrusion direction.

**Figure 5:**
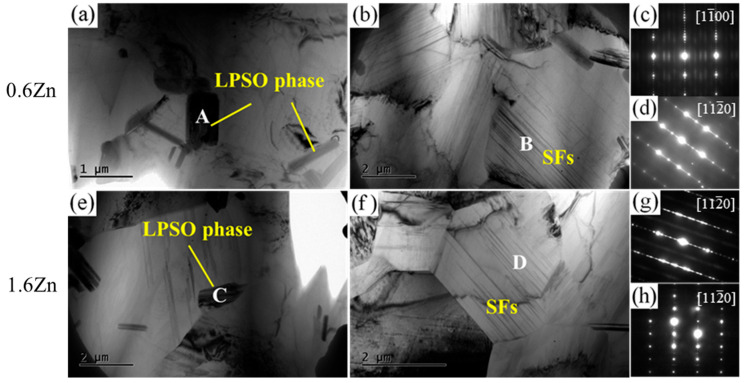
TEM bright-field images of the fine particles and lamellar phases and the corresponding SAED patterns for (**a**,**e**) fine particles and (**b**,**f**) lamellar phases. (**c**,**d**,**g**,**h**) reveal the corresponding SAED patterns of sections A, B, C, and D, respectively. The electron beam is parallel to [1$\overset{-}{1}$00]_α_ (**c**) and [11$\overset{-}{2}$0]_α_ (**d**,**g**,**h**).

In order to investigate the fine particles and lamellar phases, TEM analyses were performed on the two samples. As shown in Figure 5a,b, the fine second phases are primarily in bulk form and are mainly distributed near the grain boundaries. They do not share any specific orientation relationship with the α-Mg phase. According to the corresponding SAED patterns in Figure 5c,d, the bulk phase A and lamellar phase B, respectively, correspond to the 14H-LPSO phase and SFs. Similar to this, the corresponding SAED patterns (Figure 5g,h) of the fine bulk phase C and lamellar phase D in the 1.6Zn alloy reveal that they correspond to the LPSO phase and SFs, respectively.

### 3.2. Mechanical Properties of the As-Extruded Alloys

**Figure 6:**
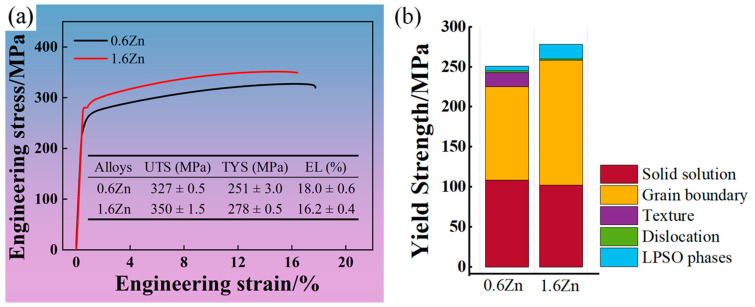
The mechanical properties of the two alloys at ambient temperature: (**a**) engineering stress–strain curves; (**b**) the contribution of each strengthening factor to the yield strength of the two alloys.

The mechanical properties of the two alloys at ambient temperature are shown in Figure 6. As depicted in Figure 6a, the 0.6Zn sample exhibited a tensile yield stress (TYS) of 251 MPa, an ultimate tensile stress (UTS) of 327 MPa, and an elongation to failure (EL) of 18.0%. The 1.6Zn sample exhibited higher strength but lower plasticity, with a TYS of 278 MPa, a UTS of 350 MPa, and an EL of 16.2%. Moreover, there is an obvious yield plateau on the tensile curve of the 1.6Zn alloy. Generally, the yield phenomenon occurs during the process of the tensile deformation of steel. This is often caused by the obstruction of dislocation slip by hard phases such as Cottrell clouds, carbonitride precipitated particles, and high-density dislocations. The authors suggest that the existence of a yield platform in the alloy is strongly correlated with a significantly higher concentration of solute atoms [31] and the ordered distribution of solute atoms in the LPSO phase. When the dislocation reached solute-rich regions in the LPSO phase, the ordered RE and Zn atoms could exert a strong pinning effect on the dislocation movement, leading to an increase in yield strength. And when dislocations broke away from the solute cluster, the resistance decreased. As a result, a yield plateau appeared on the tensile curve.

### 3.3. Fracture of the As-Extruded Alloys

**Figure 7:**
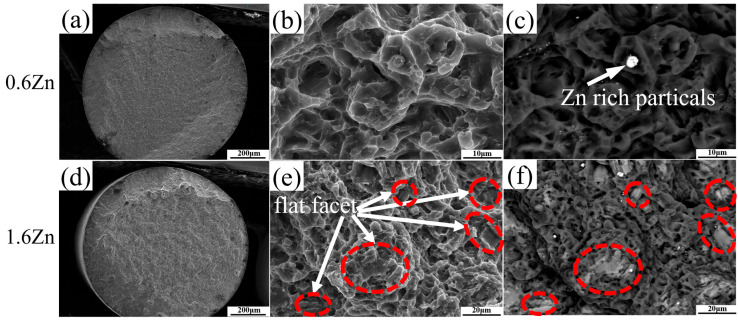
SEM micrographs of fracture surfaces for both alloys: (**a**,**d**) whole fracture; (**b**,**e**) enlarged local fractures in the sample center; (**c**,**f**) the corresponding backscattered-electron images of (**b**,**e**), respectively.

Figure 7 shows the morphology of the fracture surface for both alloys. Clear regions distinguishing the propagation and final failure stages can be observed in the two alloys, as shown in Figure 7a,d. This indicates that the alloys underwent significant plastic deformation prior to fracture. And the many dimples on the fracture surface, as shown in Figure 7b,e, further indicate that both alloys were predominantly affected by ductile fractures. However, the LPSO phases in the 1.6Zn alloy are primarily brittle, as evidenced by the well-defined facets, as shown in the red circles in Figure 7e,f.

**Figure 8:**
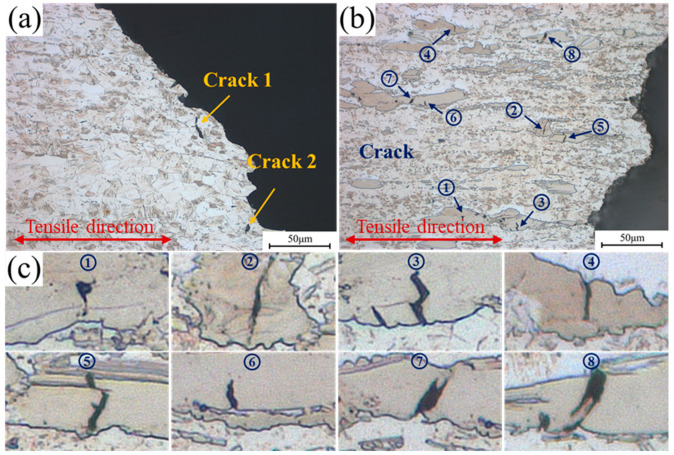
Observation of the planes perpendicular to the fractured tensile specimen surfaces reveals (**a**) cracks in the 0.6Zn specimen and that ((**b**,**c**)) LPSO phase cracking was the main fracture mechanism in the 1.6Zn specimen. (**c**) shows the enlarged photos of typical cracks ①–⑧ in (**b**). The tensile direction is horizontal.

Optical images taken near the fractures in the two alloys are shown in Figure 8a and b, respectively. The cracks in the 0.6Zn alloy are all transcrystalline cracks, while those in the 1.6Zn alloy are mainly located on the LPSO phases. Moreover, the number of cracks in the 0.6Zn alloy is significantly lower than that in the 1.6Zn alloy. The typical cracks near the fracture in the 1.6Zn alloy are summarized in Figure 8c. Except for a few cracks originating in the bulk LPSO phases, most of the cracks formed at the interface and propagated into the interior of the bulk LPSO phases. The directions of cracks are not parallel or perpendicular to the tensile direction but positioned at certain angles. Moreover, a zigzag crack was found, indicating that the direction of crack propagation changed due to stress concentration at the crack tip. This change in direction might have resulted in greater deformation resistance [33].

## 4. Discussion

The yield strength of the two alloys was determined by various strengthening factors, including solid solutes, grain boundaries, texture, dislocations, and LPSO phases. This made it difficult to accurately determine the specific contribution of LPSO phases. Consequently, it was necessary to calculate each strengthening factor that affects strength. The formula for quantitatively calculating the effect of LPSO phases on yield strength is as follows:

(1) $$\sigma_{\mathrm{LPSO}}=\sigma_{y}-\sigma_{\mathrm{ss}}-\sigma_{\mathrm{gb}}-\sigma_{\mathrm{tex}}-\sigma_{\mathrm{dis}}$$

where $\sigma_{y}$ is the yield strength, and $\sigma_{LPSO}$, $\sigma_{ss}$, $\sigma_{gb}$, $\sigma_{tex}$, and $\sigma_{dis}$ are the strengthening contributions of LPSO phases, solid solution, grain boundaries, texture, and dislocations, respectively. Next, we will analyze and quantitatively calculate the contribution of each strengthening factor on the right side of Equation (1) to strength and clarify the strengthening effect of the LPSO phases.

### 4.1. Solution Strengthening

According to Ref. [34], the composition of LPSO phases in the two alloys is Mg_12_REZn, in which the sum of Gd and Y is equal to Zn. Since both Gd and Y can participate in the formation of LPSO phases and replace each other in LPSO phases [27], we assume that the ratio of Gd to Y in LPSO phases is consistent with that in the alloys, as shown in Table 1. Then, the atomic percentages of different solutes in the α-Mg grains could be approximately calculated based on the volume fractions of LPSO phases, which were measured to be 0.23% in the 0.6Zn alloy and 4.71% in the 1.6Zn alloy, according to the quantitative statistics obtained from their SEM micrographs. Further, the atomic percentages of alloy elements in α-Mg grains in the two alloys were calculated, and they are presented in Table 2. It should be noted that the Gd, Y, and Zn atoms within the interior of the lamellar phases in the grains also need to be considered. In our previous research [29], we found that when basal slip is predominant, the strengthening effect of Gd, Y, and Zn atoms in lamellar phases on the alloy is slightly less than that in solid solution. In other words, the precipitation of lamellar LPSO phases in the α-Mg grains will slightly decrease the strength of the alloys. The calculated reduced value is approximately 7 MPa. The number of intragranular lamellar phases precipitated during or after extrusion is theoretically less for the two alloys in this case compared to those precipitated during furnace cooling and preservation after homogenization. And they mainly occur in the DRXed grains with relatively random orientations. There is no significant difference in the content of lamellar phases between the two alloys. That is, the strength reduction caused by the precipitation of lamellar phases in both alloys was essentially identical, and both reductions are minimal. Considering that we are finally about to compare the influences of strengthening factors on the properties of the two alloys, we will not take into account the influence of lamellar precipitation in this calculation. And we assume that the Gd, Y, and Zn atoms are all dissolved into the Mg grains, except for those in bulk LPSO phases, in subsequent calculations. However, for the sake of precision, we still need to note that there may be a potential error in the calculated strength difference, with an absolute value ≤ 7 MPa, because it might affect our subsequent analysis of the contribution of bulk LPSO phases.

Further, if we assume that Gd, Y, and Zn atoms are present together without interacting with each other in the α-Mg solute, the strengthening effect resulting from multiple alloying additions in the Mg-Gd-Y-Zn alloys can be determined using the formula proposed by Gypen and Deruyttere [35]. This formula has been proven effective in predicting the yield strength of solid-solution alloys for a series of experimental and commercial nickel-based alloys [35] and Mg-Gd-Y alloys [36]; it is shown below

(2) $$\sigma_{\mathrm{ss}}={\left(\sum k_{i}^{1/n}c_{i}\right)}^{n}$$

where n is a constant, $c_{i}$ is the concentration of solute i, and $k_{i}$ is the strengthening constant for solute i. Then, for Mg-Gd-Y-Zn alloys, it is written as

(3) $$\sigma_{\mathrm{ss}}={\left(k_{Gd}^{1/n}c_{Gd}+k_{Y}^{1/n}c_{Y}+k_{Zn}^{1/n}c_{Zn}\right)}^{n}$$

where $c_{Gd}$, $c_{Y}$, and $c_{Zn}$ are the atomic fractions of solutes; $k_{Gd}$ (1168 MPa (at.%)^−2/3^), $k_{Y}$ (1249 MPa (at.%)^−2/3^) [36], and $k_{Zn}$ (432 MPa (at.%)^−2/3^) [37] are the alloy strengthening rates; and n is a constant, taken as 2/3 in the Mg-Gd-Y series alloys indicated by the critical examination [36]. Thus, the strength increment for 0.6Zn and 1.6Zn due to solution strengthening could be calculated as 108.8 MPa and 102.2 MPa, respectively.

### 4.2. Grain Boundary Strengthening

Grain refinement is recognized as one of the most effective methods for improving strength and plasticity simultaneously. Grain boundary strengthening can be calculated by making use of the Hall–Petch law [38]:

(4) $$\sigma_{gb}=\sigma_{0}+kd^{-1/2}$$

where $\sigma_{0}$ is the intrinsic lattice strength (36.0 MPa [39]), and k is the influence coefficient of grain boundary on deformation, which has been reported to equal 240 MPa·μm^−1/2^ [40,41]. Then, the strength increments of the 0.6Zn and 1.6Zn alloys due to grain boundary strengthening were calculated, equaling 116.4 MPa and 156.0 MPa, respectively.

### 4.3. Texture Strengthening

At room temperature, basal slip ({0001}<1120>) is the dominant deformation mechanism for magnesium alloys containing LPSO phases [41]. The Schmid factor (SF) of the {0001}<1120> basal slip system [42] corresponds to the texture results. According to the statistical results regarding SFs in Figure 4, the average SF for the alloy 0.6Zn is 0.26, while that for the 1.6Zn alloy is 0.30. In this case, texture strengthening [43] can be expressed using the following equation:

(5) $$\sigma_{tex}=(0.3/\mathrm{SF}-1)\times (\sigma_{0}+kd^{-1/2})$$

Then, the contributions of texture strengthening for 0.6Zn and 1.6Zn were calculated to be 17.9 MPa and 0 MPa, respectively.

### 4.4. Dislocation Strengthening

Dislocation strengthening $\sigma_{dis}$ mainly occurs due to the presence of dislocations in grains, while DRXed grains are not considered due to their significantly lower dislocation density compared to unDRXed grains. Dislocation strengthening can be calculated using the following equation [44]:

(6) $$\sigma_{dis}=f_{unDRX}M_{unDRX}\alpha Gb\sqrt{\rho_{unDRX}}$$

where *α* is a constant (0.2 for Mg [45]), *G* is the shear modulus (16.6 GPa for Mg [46]), *b* is the Burger’s vector value (3.21 × 10^−10^ m for Mg [46]), *M* is the Taylor factor for un-DRXed regions (3.5 for Mg with a strong texture [45]), $\rho_{unDRX}$ is the dislocation density in un-DRXed regions (a typical value of 1 × 10^14^ m^−2^ for deformed Mg alloys [44]), and $f_{unDRX}$ represents the volume fractions of un-DRXed regions, as determined via EBSD analysis, which were found to be 6.2% and 4.0% for 0.6Zn and 1.6Zn, respectively. As a result, the strength increments of the 0.6Zn and 1.6Zn alloys due to dislocation strengthening were calculated to be 2.3 MPa and 1.5 MPa, respectively, indicating that they contribute little to the yield strength.

### 4.5. The Role of Bulk LPSO Phases

The strength increments induced by the above strengthening factors are summarized in Table 3. By incorporating these values into Equation (1), it was easy to calculate that the contributions of LPSO phases to the strength of the 0.6Zn alloy and 1.6Zn alloy are equal to 5.6 MPa and 18.3 MPa, respectively. The contributions of each strengthening factor to the yield strength of the two alloys are summarized in Figure 6b. Considering the relatively low occurrence of lamellar LPSO phases in the as-extruded alloys, as well as their limited strengthening effect in the DRXed grains with random orientations, where basal dislocations are more likely to be induced [14,29], the difference in strengthening provided by the lamellar LPSO phases in the two alloys is disregarded here. Note that some submicron-sized bulk LPSO phases may appear near grain boundaries after extrusion, similar to the situation for the 1.6Zn alloy. Obviously, these LPSO phases play a strengthening role in the 0.6Zn alloy. For the 1.6Zn alloy, in addition to these submicron-sized LPSO phases, the presence of coarse bulk LPSO phases also contributes to strengthening.

LPSO phases are often considered beneficial for enhancing plasticity because they can facilitate the coordinated plastic deformation of alloys through dislocation slip and kinking. The presence of zigzag cracks on LPSO phases, as observed in this study, indicates their superior toughness compared to the typically hard and brittle phases found in magnesium alloys. However, it still needs to be emphasized that the existence of bulk LPSO phases is detrimental to plasticity. This is based on the fact that the plasticity of the 1.6Zn alloy is slightly lower than that of the 0.6Zn alloy, despite the improvement in ductility due to grain refinement and weakening of the basal texture in this alloy.

**Figure 9:**
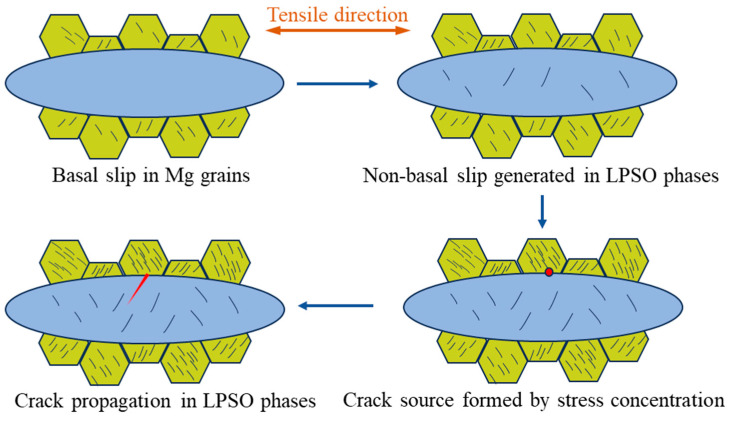
A schematic diagram of coordinated plastic deformation and cracking of the bulk LPSO phase, in which the dots or wedges marked in red represent crack origination or expansion, respectively.

In fact, the superior plasticity of high-performance Mg-RE-Zn alloys is generally considered to be derived from the comprehensive effects of LPSO phases, fine grains, a weaker texture, etc. Among them, LPSO phases are assumed to be beneficial to plasticity, mostly based on their plastic behavior, such as kinking during deformation. However, their true impact on final ductility is not clear. Recently, Takagi et al. [24] reported a single 18-LPSO crystal with remarkable ductility and a final failure at ε ≈ 70%, clearly affirming the excellent plastic deformation ability of LPSO phases. However, when it comes to polycrystalline and multiphase Mg-RE-Zn alloys, it is important to take into account not only the coordinated deformation ability of the LPSO phases but also the deformation incompatibility between the LPSO phases and α-Mg phases. According to the study conducted by Egusa [11], ABCA-type units in LPSO phases can be considered to be a consequence of a severely distorted lattice caused by the accumulation of RE and Zn solute atoms [11]. This contrasts with the ideal ABCA stacking found in aluminum alloys. As a result, bulk LPSO phases demonstrate significantly greater stiffness than α-Mg [47]. Furthermore, during the hot extrusion process, bulk LPSO phases undergo severe plastic deformation and even crushing, while α-Mg is softened by dynamic recrystallization. This results in the LPSO phases being significantly harder than the α-Mg phases. Thus, the plastic deformation ability of LPSO phases decreases, and the discrepancy in deformation between LPSO phases and α-Mg further increases. Therefore, although bulk LPSO phases might be able to coordinate the deformation of the α-Mg phases through their own deformation, allowing them to contribute more to plasticity than the generally brittle phase, stress concentration would still easily occur on the LPSO phases due to their weakness in continuously coordinating deformation, resulting in microcracks. Thus, even if crack propagation in the LPSO phases absorbs some energy, it cannot prevent the premature fracture of the alloy. A schematic diagram illustrating the coordinated plastic deformation and cracking of the bulk LPSO phase is presented in Figure 9.

By analyzing the crack positions in Figure 8, it was observed that the cracks mainly appear in three types of positions: (1) at the junction of the interface formed due to kinking after severe the deformation of the LPSO phases, such as positions ① and ②; (2) at the step position induced by the stratification of LPSO phases due to severe deformation, such as positions ③ and ⑤; and (3) at the sharp concave tips of the LPSO phases, such as positions ④, ⑥, ⑦, and ⑧. Among them, the first two types of cracking are related to defects in the bulk LPSO phases. These defects are intense deformation areas and interfaces that are generated inside the deformed LPSO phases due to severe plastic deformation during extrusion. Due to the significant decrease in the deformation ability of hardened LPSO phases, further stress concentration inside these phases can lead to cracking during tensile deformation at room temperature. The third type of cracking, located at the interface between α-Mg and LPSO phases, was attributed to deformation incompatibility between the LPSO phases and α-Mg phases. It is worth noting that the cracks in the LPSO phases did not propagate quickly or cause damage to the 1.6Zn alloy. Instead, they appeared within various forms in the LPSO phases. Consequently, the 1.6Zn alloy still maintained excellent plasticity, even with these cracks in the LPSO phases. The stress release caused by cracking in the LPSO phases competes with the work hardening in the non-crack area, and no corresponding softening phenomenon can be observed in the stress–strain curve.

In summary, bulk LPSO phases can strengthen alloys by refining the grains during deformation and acting as strengthening phases. However, they can also lead to the premature fracture of the 1.6Zn alloy due to limited coordinated deformation ability. Therefore, it is necessary to consider whether the current form of LPSO phases can be further optimized to enhance both strength and plasticity.

### 4.6. A Design Strategy for High-Performance Mg-RE-Zn Alloys

In order to determine the most effective LPSO phase forms for superior strengthening and toughening effects, we conducted a study on the lamellar LPSO phases in α-Mg grains predominantly affected by basal dislocation slip [29], as well as bulk LPSO phases, in this research. We analyzed their influence on strength and plasticity and identified any existing issues.

Eventually, a design strategy for incorporating LPSO phases into high-strength and high-plasticity Mg-RE-Zn alloys was proposed. Firstly, considering the significant solid-solution-strengthening effect of RE and Zn, as well as their subsequent aging-strengthening effect, it is necessary to retain the solute atoms of RE and Zn in the α-Mg phases. Secondly, considering the significant contribution of fine-grain refinement to strength and its benefits for plasticity, further refining the grain size would be a very effective approach. This can be achieved through large plastic deformation or low-temperature forging. Thirdly, because the large size of the bulk LPSO phases makes it difficult to coordinate deformation after work hardening, which deteriorates plasticity, it is better to refine or even eliminate bulk LPSO phases. Finally, due to the orientation of the lamellar LPSO phases within the grains being parallel to the basal plane of the α-Mg phase, they are not effective in hindering basal plane slip. However, they show potential in inhibiting slip on non-basal planes [48,49]. Therefore, promoting slip in non-basal planes would be more conducive to enhancing the strengthening effect of the lamellar LPSO phases. Accordingly, there are two effective methods for achieving this. First, the texture can be constructed in a way that discourages basal slip in a specific direction, that is, reducing the Schmidt factor of basal slip. This can be achieved through deformation and other processes, such as creating a basal plane texture parallel to the extrusion direction and applying loading along the extrusion direction. These processes promote the occurrence of non-basal plane slip. Additionally, a specific form of precipitation phases (preferably columnar, prismatic, disc-shaped, and perpendicular to the basal plane) can be created between the lamellar LPSO phases through aging treatment or other methods, such as the formation of a β1 phase and a β′ phase during the aging treatment at 200~300 °C. In general, the prudent strategy is to leverage the toughening characteristics of LPSO phases while maintaining the high-strength benefits of Mg-RE alloys in order to develop high-performance magnesium alloys that possess both high strength and high plasticity.

## 5. Conclusions

In this work, to investigate the impact of bulk LPSO phases on mechanical properties and fracture behaviors, the Mg-8Gd-4Y-0.6Zn-0.5Zr alloy, without LPSO phases, and Mg-8Gd-4Y-1.6Zn-0.5Zr alloy, containing LPSO phases, were extruded, and their microstructures and mechanical properties have been comparatively analyzed. The following conclusions were drawn:

1. The bulk LPSO phases promote grain refinement and weaken the texture during the plastic deformation process for the 1.6Zn alloy, constituting effects that are beneficial to plasticity. Additionally, in this way, the LPSO phases significantly contribute to the strength of the 1.6Zn alloy due to the significant strengthening effect of grain refinement, notwithstanding the modest reduction in strength caused by the weakening of texture.
2. Attributed to the high Young’s modulus and shear modulus of bulk LPSO phases, as well as their severe deformation after extrusion, bulk LPSO phases act as hard phases, and this directly contributes to the strength increment. However, due to the fact that the bulk LPSO phases after extrusion could not effectively coordinate deformation and easily crack due to stress concentration, bulk LPSO phases themselves play a negative role in plasticity during tensile deformation and cause the premature fracturing of the 1.6Zn alloy.
3. A strengthening strategy intended to maintain very good solid-solution strengthening and fine-grain strengthening, optimize the size and distribution of the bulk LPSO phase, and enhance the interaction of the lamellar LPSO phase with the non-basal dislocations was proposed.

## Figures and Tables

**Table 1:** The chemical compositions of the studied alloys analyzed via ICP-AES.

Alloys	Gd (wt.%/at.%)	Y (wt.%/at.%)	Zn (wt.%/at.%)	Zr (wt.%/at.%)	Mg (wt.%/at.%)
0.6Zn	8.60/1.49	3.88/1.19	0.66/0.28	0.50/0.15	Balance
1.6Zn	8.51/1.49	4.03/1.24	1.60/0.67	0.49/0.15	Balance

**Table 2:** Content of solid-solution elements in extruded α-Mg grains.

Alloys	Volume Fraction of LPSO Phases	Content of Alloy Elements in α-Mg Grains/at.%		
		Gd	Y	Zn
0.6Zn	0.23%	1.48	1.18	0.26
1.6Zn	4.71%	1.31	1.09	0.33

**Table 3:** The calculated contributions of different factors to the yield strength.

Alloys	$\sigma_{\mathrm{ss}}$/MPa	$\sigma_{gb}$/MPa	$\sigma_{tex}$/MPa	$\sigma_{dis}$/MPa
0.6Zn	108.8	116.4	17.9	2.3
1.6Zn	102.2	156.0	0	1.5

## Data Availability

The raw/processed data required to reproduce these findings cannot be shared at this time due to technical or time limitations, but they can be made available by the corresponding author upon request.
